# Ergosterol Peroxide from the Medicinal Mushroom *Ganoderma lucidum* Inhibits Differentiation and Lipid Accumulation of 3T3-L1 Adipocytes

**DOI:** 10.3390/ijms21020460

**Published:** 2020-01-10

**Authors:** Yong-Un Jeong, Young-Jin Park

**Affiliations:** 1 Department of Medicinal Bioscience, College of Biomedical and Health Science, Konkuk University, 268 Chungwon-daero, Chungju-si 27478, Korea; s11034@kku.ac.kr; 2 Research Institute for Biomedical & Health Science, Konkuk University, 268 Chungwon-daero, Chungju-si 27478, Korea

**Keywords:** adipocyte, differentiation, ergosterol peroxide, *Ganoderma lucidum*, lipid, mushroom

## Abstract

Ergosterol peroxide is a natural compound of the steroid family found in many fungi, and it possesses antioxidant, anti-inflammatory, anticancer and antiviral activities. The anti-obesity activity of several edible and medicinal mushrooms has been reported, but the effect of mushroom-derived ergosterol peroxide on obesity has not been studied. Therefore, we analyzed the effect of ergosterol peroxide on the inhibition of triglyceride synthesis at protein and mRNA levels and differentiation of 3T3-L1 adipocytes. Ergosterol peroxide inhibited lipid droplet synthesis of differentiated 3T3-L1 cells, expression of peroxisome proliferator-activated receptor gamma (PPARγ) and CCAT/enhancer-binding protein alpha (C/EBPα), the major transcription factors of differentiation, and also the expression of sterol regulatory element-binding protein-1c (SREBP-1c), which promotes the activity of PPARγ, resulting in inhibition of differentiation. It further inhibited the expression of fatty acid synthase (FAS), fatty acid translocase (FAT), and acetyl-coenzyme A carboxylase (ACC), which are lipogenic factors. In addition, it inhibited the phosphorylation of mitogen-activated protein kinases (MAPKs) involved in cell proliferation and activation of early differentiation transcription factors in the mitotic clonal expansion (MCE) stage. As a result, ergosterol peroxide significantly inhibited the synthesis of triglycerides and differentiation of 3T3-L1 cells, and is, therefore, a possibile prophylactic and therapeutic agent for obesity and related metabolic diseases.

## 1. Introduction

Obesity is one of the most common diseases worldwide [1]. According to the WHO, obesity worldwide has increased by nearly three times since 1975. It is reported that 39% of the world’s adult population is overweight, and 13% or more of 650 million adults are obese [2]. Because obesity is associated with many diseases including type 2 diabetes, high blood pressure, cancer and stroke [3], its prevention and treatment are very important. Obesity is caused by excessive accumulation of fatty tissue as a result of an imbalance between energy intake and energy consumption. The increase in adipose tissue is caused by an increase in the number of differentiated adipocytes and an increase in the size of adipocytes due to lipid accumulation. Therefore, inhibiting the proliferation and differentiation of adipocytes may be an effective method for the prevention or treatment of obesity and related metabolic diseases [4].

Several animal (3T3-L1, 3T3-F442A, C3H10T1/1, OP9, MEFs, porcine primary preadipocytes, and feline primary preadipocytes) and human (adipose-derived stem cells, primary preadipocyte, and adipocytes) cell models are currently available for studying the process of in vitro adipogenesis [5]. Moreover, co-cultures and three-dimensional (3D) cultures system of adipocytes with other cell types are also available to understand metabolic interactions between adipocytes and other cells [6]. Among them, 3T3-L1 cells were presented by Howard Green in 1974 and are commonly used as a cell model to study differentiation in adipocytes [7]. Differentiation from preadipocytes to adipocytes consists of three stages: growth arrest, increase by somatic cell replication, and differentiation [8]. The cell cycle of preadipocytes, which have ceased growth by cell conjugation, is activated by cell differentiation-inducing media containing IBMX (3-isobutyl-1-methylxanthine), dexamethasone, and insulin. Increasing cell numbers by cell division requires activation of the mitogen-activated protein kinases (MAPKs) pathway, including extracellular signal-regulated kinase (ERK), c-jun N-terminal kinase (JNK), and p38 [9,10,11]. This process also activates several early fat-producing transcription factors, including CCAT/enhancer-binding protein beta (C/EBPβ) and CCAT/enhancer-binding protein delta (C/EBPδ) [12]. These transcription factors stimulate the peroxisome proliferator-activated receptor gamma (PPARγ) and CCAT/enhancer-binding protein alpha (C/EBPα), which are essential transcription factors mediating the differentiation process [12]. Expressed PPARγ and C/EBPα interact with each other, and each promotes the expression of the other. Although the independent roles of each have not been clearly identified, it is commonly accepted that PPARγ’s role is more dominant [13]. The interaction of PPARγ and C/EBPα is known to induce the expression of adipocyte-specific adipokines including fatty acid synthase (FAS), stearoyl-coenzyme A desaturase 1 (SCD-1), acetyl-coenzyme A carboxylase (ACC), and fatty acid translocase (FAT) [14,15,16]. In addition, the transcription factor sterol regulatory element-binding protein-1c (SREBP-1c) has also been reported to positively regulate adipocyte differentiation [17].

In a screening of triacylglycerol synthesis inhibitors from natural products, *G. lucidum* was selected as a natural resource. *G. lucidum* has been used for medicinal purposes for centuries, particularly in China, Japan, and Korea. It has been for the treatment of migraine hypertension, diabetes, hypercholesterolaemia, and cardiovascular problems. In addition, it was reported that *G. lucidum* extract showed hypoglycemic activity by increasing plasma insulin and by affecting hepatic enzymes in alloxan-induced diabetic mice [18,19,20]. However, *G. lucidum* extract is frequently prescribed in combination for synergistic effects or to diminish possible adverse reactions. At present, the chemical constituents and bioactivities of the fruiting bodies of *G. lucidum* have been fully investigated, and the triterpenoids were found to be the most important active substances for its numerous pharmacological uses [21]. More than 100 triterpenes and steroids have been identified from *G. lucidum* [22]. Among them is ergosterol peroxide (5α, 8α-epidioxy-22*E*-ergosta-6,22-dien-3β-ol), a natural steroid that has been found in a variety of fungi [22,23,24] and is synthesized by the H_2_O_2_-dependent enzymatic oxidation of ergosterol. Ergosterol peroxide has been shown to inhibit inflammation [25] and cancer cell proliferation [26], as well as induce apoptosis of cancer cells [27,28,29]. Ergosterol peroxide was first isolated from the genus *Aspergillus* in 1947 [30] and is reported to be found in various organisms, including algae, lichens, corals, and mushrooms [31,32,33,34,35]. In addition, several kinds of mushroom fruiting bodies or mycelium extracts, including *Ganoderma lucidum*, *G. applanatum*, *Phellinus linteus*, and *Cordyceps militaris*, have been reported to suppress obesity and related metabolic diseases [36,37,38,39]. However, studies on the inhibition of obesity or differentiation of adipocytes by ergosterol peroxide are insufficient. Therefore, the purpose of this study is to investigate the possible use of ergosterol peroxide from the medicinal mushroom *Ganoderma lucidum* as a bioactive substance for the prevention or treatment of obesity by inhibiting 3T3-L1 cell differentiation and triglyceride synthesis. Here, we report the first results demonstrating that ergosterol peroxide present in the medicinal mushroom *G*. *lucidum* is a potent agent for regulating abnormal fat metabolism.

## 2. Results 

### 2.1. Chemical Structure and Cytotoxicity of Ergosterol Peroxide on 3T3-L1 Cells

**Figure 1:**
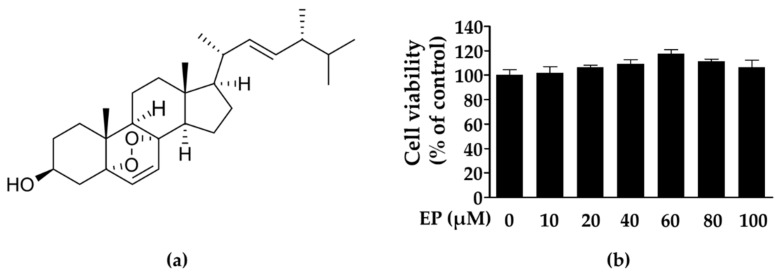
Molecular structure (**a**) and cytotoxic effects (**b**) of ergosterol peroxide isolated from *G. lucidum* on 3T3-L1 cells. 3T3-L1 cells were treated with various concentration of ergosterol peroxide (10, 20, 40, 60, 80, and 100 μM) for 48 h. The values are expressed as mean ± standard deviation of independent experiments performed in triplicate. EP: ergosterol peroxide.

Initially, the ethanol extract of *G. lucidum* was suspended in water and partitioned with ethyl acetate. Using bioassay-guided fractionation, the ethyl acetate fraction was separated by column chromatography to obtain ergosterol peroxide. We compared the isolated ergosterol peroxide with spectroscopic nuclear magnetic resonance (NMR) data previously reported in the literature (Figure 1a) [40]. Ergosterol peroxide (5α, 8α-epidioxy-22*E*-ergosta-6, 22-dien-3β-ol) showed the following characteristics: colorless needles, C_28_H_44_O_3_; ^1^H-NMR (CDCl_3_, 300 MHz): δ 0.81 (3H, s, H-18), 0.82 (3H, d, *J* = 4.5 Hz, H-26), 0.83 (3H, s, H-27), 0.88 (3H, s, H-19), 0.90 (3H, d, *J* = 6.6 Hz, H-28), 0.99 (3H, d, *J* = 6.6 Hz, H-21), 3.96 (1H, m, H-3), 5.13 (1H, dd, *J* = 8.1, 15 Hz, H-22), 5.21 (1H, dd, *J* = 7.5 Hz, 15.36 Hz H-23), 6.24 (1H, d, *J* = 8.4 Hz, H-6), 6.51 (1H, d, *J* = 8.4 Hz, H-7). ^13^C-NMR (75 MHz, CDCl_3_): δ 12.84 (C-18), 17.53 (C-28), 18.15 (C-19), 19.61 (C-27), 19.92 (C-26), 20.60 (C-15), 20.85 (C-21), 23.37 (C-11), 28.61 (C-16), 30.08 (C-2), 33.04 (C-25), 34.67 (C-1), 36.89 (C-10), 36.94 (C-4), 39.32 (C-12), 39.7 (C-20), 42.75 (C-24), 44.53 (C-13), 51.06 (C-9), 51.65 (C-14), 56.17 (C-17), 66.43 (C-3), 79.40 (C-8), 82.13 (C-5), 130.72 (C-7), 132.28 (C-23), 135.17 (C-22), 135.39 (C-6).

We examined the cytotoxic effects of ergosterol peroxide on 3T3-L1 cells treated with the indicated concentrations (10, 20, 40, 60, 80, and 100 μM) for 48 h. As shown in Figure 1b, ergosterol peroxide showed no cytotoxic effects on 3T3-L1 cells in the MTT assay. Therefore, in this study, additional experiments were carried out using 20 μM to maintain cell viability following repetitive treatments for differentiation. 

### 2.2. Effect of Ergosterol Peroxide on Lipid Droplet Synthesis in 3T3-L1 Cells

**Figure 2:**
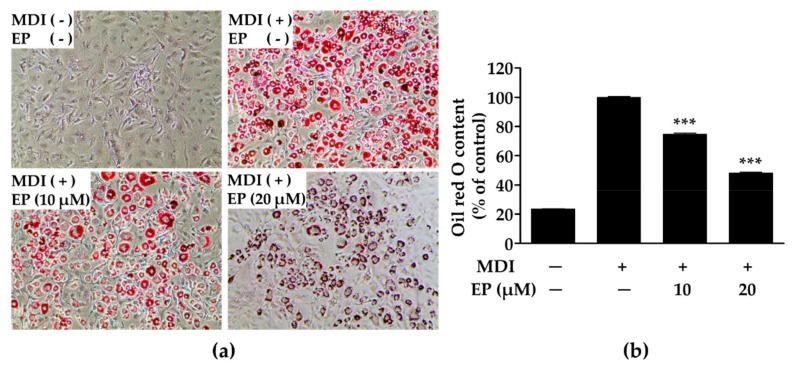
Microscopic morphologies of 3T3-L1 cells stained with Oil red O (**a**) and quantification of lipid droplet (**b**). 3T3-L1 cells were stained with Oil red O, and then photographed under microscope (Magnification: 100×). The values are expressed as mean ± standard deviation of independent experiments performed in triplicate. The data was analyzed using one-way analysis of variance (ANOVA) followed by Dunnett’s test. *** *p* < 0.001 versus differentiated 3T3-L1 cells without ergosterol peroxide treatment. MDI: methylisobutylxanthine, dexamethasone, and insulin; EP: ergosterol peroxide.

As shown in Figure 2, ergosterol peroxide inhibited lipid droplet synthesis. In untreated 3T3-L1 cells, no lipid droplets were observed, whereas a large amount of lipid droplets were observed in MDI-treated (methylisobutylxanthine, dexamethasone and insulin) cells (Figure 2a). However, MDI-treated cells incubated with ergosterol peroxide at concentrations of 10 and 20 μM showed significantly lower quantities of lipid droplets (Figure 2b) than untreated cells. Importantly, the inhibitory effect of ergosterol peroxide was not due to cytotoxicity, as cell viability did not decrease in the presence of ergosterol peroxide (80 µM; Figure 1b). These results suggest that ergosterol peroxide from *G. lucidum* can reduce the accumulation of lipid droplets by repressing adipogenesis. 

### 2.3. Effect of Ergosterol Peroxide on Adipogenic Transcription Factors in 3T3-L1 Cells

**Figure 3:**
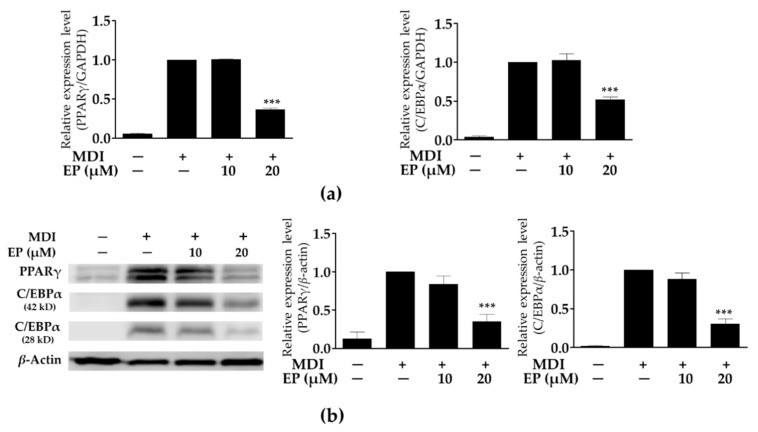
Effect of ergosterol peroxide on peroxisome proliferator-activated receptor gamma (PPARγ) and CCAAT/enhancer binding protein alpha (C/EBPα) mRNA transcription (**a**) and protein expression (**b**) in 3T3-L1 cells. The mRNA transcription and protein expression levels were normalized glyceraldehyde-3-phosphate dehydrogenase (GAPDH) and β-actin expression levels, respectively, as an internal reference. The values are expressed as mean ± standard deviation of independent experiments performed in triplicate. The data was analyzed using one-way analysis of variance (ANOVA) followed by Dunnett’s test. *** *p* < 0.001 versus differentiated 3T3-L1 cells without ergosterol peroxide treatment. EP: ergosterol peroxide.

The transcription of PPARγ and C/EBPα mRNA was significantly increased in MDI-treated cells compared to undifferentiated cells. As shown in Figure 3a, treatment with 20 μM of ergosterol peroxide significantly reduced PPARγ and C/EBPα mRNA transcription levels in MDI-treated cells; however, treatment with 10 μM did not suppress their transcription levels. Likewise, expression of PPARγ and C/EBPα proteins was decreased by treatment with ergosterol peroxide in MDI-treated cells in a dose-dependent manner. Moreover, treatment with 20 μM of ergosterol peroxide significantly reduced PPARγ and C/EBPα protein expression levels compared to the undifferentiated cells (Figure 3b). These results indicate that treatment with ergosterol peroxide from *G. lucidum* contributes to the reduction in PPARγ and C/EBPα protein expressions through the suppression of mRNA transcription.

### 2.4. Effect of Ergosterol Peroxide on Adipokines in 3T3-L1 Cells

**Figure 4:**
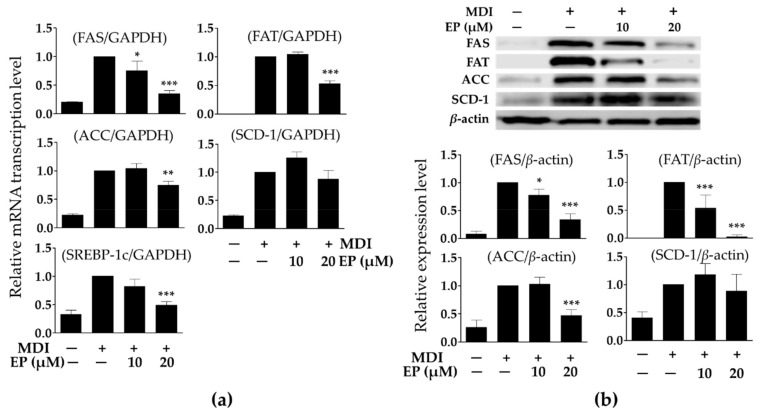
Effect of ergosterol peroxide on transcription of adipokines (**a**) and protein expression (**b**) in 3T3-L1 cells. The mRNA transcription and protein expression levels were normalized to glyceraldehyde-3-phosphate dehydrogenase (*GAPDH*) and *β-actin* expression levels, respectively, as an internal reference. Relative protein expression levels were analyzed by Image J software (National Institutes of Health, Bethesda, MD, USA). The values are expressed as mean ± standard deviation of independent experiments performed in triplicate. The data were analyzed using one-way analysis of variance (ANOVA) followed by Dunnett’s test. * *p* < 0.05, ** *p* < 0.01, and *** *p* < 0.001 versus differentiated 3T3-L1 cells without ergosterol peroxide treatment. FAS: fatty acid synthase; FAT: fatty acid translocase; ACC acetyl-CoA carboxylase; SCD-1: stearoyl-CoA desaturase-1; SREBP-1c: sterol regulatory element binding transcription factor-1c; MDI: methylisobutylxanthine, dexamethasone, and insulin; EP: ergosterol peroxide.

The expression of FAS, FAT, ACC, SCD-1, and SREBP-1c mRNA in MDI-treated cells increased significantly compared to the undifferentiated cells. As shown in Figure 4a, treatment with 20 μM of ergosterol peroxide significantly reduced FAS, FAT, ACC, and SREBP-1c mRNA expression in MDI-treated cells, but treatment with 20 μM ergosterol peroxide did not suppress SCD-1 mRNA expression. Likewise, FAS, FAT, and ACC protein expressions were significantly decreased by treatment with ergosterol peroxide (10 or 20 μM) in the MDI-treated cells (Figure 4b). These results also suggest that treatment with ergosterol peroxide from *G. lucidum* contributes to the reduction in FAS, FAT, and ACC protein expressions through the suppression of mRNA transcription.

### 2.5. Effect of Ergosterol Peroxide on Phosphorylated p38, c-Jun N-Terminal Kinase (JNK), and Extracellular Signal-Regulated Kinase (ERK) Protein Levels in 3T3-L1 Cells

**Figure 5:**
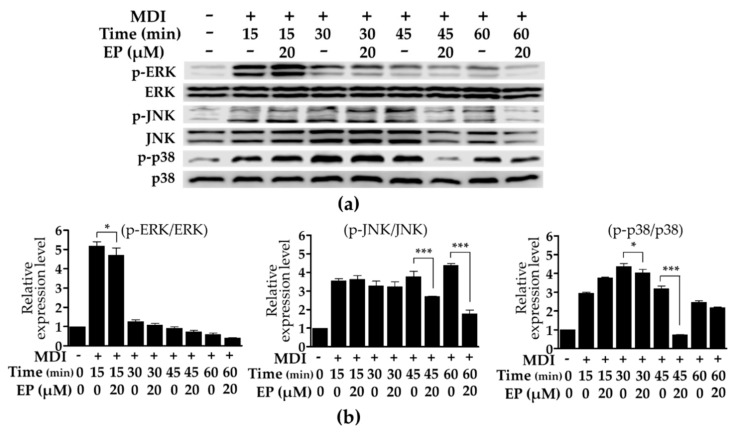
Effects of ergosterol peroxide on the phosphorylation of extracellular signal-regulated kinase (ERK), c-Jun N-terminal kinase (JNK), and p38 proteins in 3T3-L1 cells. Cellular protein levels were examined using Western blot analysis (**a**) and relative phosphorylation levels (**b**) were analyzed by Image J software (National Institutes of Health, USA). The values are expressed as mean ± standard deviation of independent experiments performed in triplicate. The data were analyzed using one-way analysis of variance (ANOVA) followed by Dunnett’s test. * *p* < 0.05 and *** *p* < 0.001 versus differentiated 3T3-L1 cells without ergosterol peroxide treatment. p-ERK: phosphorylated ERK; p-JNK: phosphorylated JNK; p-p38: phosphorylated p38; MDI: methylisobutylxanthine, dexamethasone, and insulin; EP: ergosterol peroxide.

Proteins in the mitogen-activated protein kinase (MAPK) pathway, including extracellular signal-regulated kinase (ERK), c-jun N-terminal kinase (JNK), and p38, are involved in the early stages of differentiation and adipocyte proliferation [9,10,11]. Proteins were isolated at 15 min intervals after MDI-treatment and the phosphorylation of ERK, JNK, and p38 proteins was analyzed using Western blot (Figure 5a). The phosphorylation level of ERK, JNK, and p38 proteins was increased significantly by MDI-treatment. MDI-induced phosphorylation of ERK proteins was significantly reduced at 15 min following treatment with 20 μM of ergosterol peroxide (Figure 5b). In addition, the MDI-induced phosphorylation of JNK protein was found to decrease significantly after 45 min following treatment with 20 μM of ergosterol peroxide (Figure 5b). Moreover, MDI-induced phosphorylation of p38 protein increased at 15 min following treatment with 20 μM of ergosterol peroxide, but significantly decreased at 30 and 45 min (Figure 5b). Therefore, treatment with ergosterol peroxide inhibited MDI-induced phosphorylation of ERK, JNK, and p38, which may help to inhibit triglyceride synthesis by inhibiting the MAPK pathway involved in the early stages of adipocyte differentiation.

## 3. Discussion

Ergosterol peroxide belongs to the steroidal family of natural products and is a major active compound present in *G. lucidum* [30,41,42] and other edible or medicinal mushrooms [24,25,26,29,43]. However, the other biological activities of ergosterol peroxide, including the anti-obesity activity, remain to be elucidated. Therefore, this study was conducted to investigate the anti-adipogenic effect of ergosterol peroxide.

Recently, several cell models and their culture systems have been developed and evaluated to understand in vitro adipogenesis [5]. However, the most representative cellular model of adipocyte differentiation studies is 3T3-L1 cells due to standardized protocols and extensive research on these cells [5,7]. 3T3-L1 preadipocytes differentiate into adipocytes by FBS supplementation along with MDI treatment and accumulate triglycerides inside the cells [7]. In general, preadipocyte 3T3-L1 cells accumulate lipid due to differentiation and cause hypertrophy of cells [4]. Therefore, we analyzed the effects of various concentrations of ergosterol peroxide (10, 20, 40, 60, 80, and 100 μM) on 3T3-L1 cell viability, and found that at all concentrations, ergosterol peroxide treatment did not affect cell viability. Oil red O staining analysis revealed that ergosterol peroxide effectively inhibits lipid accumulation of 3T3-L1 cells. Adipogenesis is a highly regulated process requiring coordinated expression and activation of key transcription factors such as PPAR-γ and C/EBPα [13,15,16]. The earliest events in adipocyte differentiation are characterized by increased expression of PPAR-γ and C/EBPα, the central transcriptional regulators of adipogenesis, which induce adipocyte-specific gene expression [16]. PPARγ and C/EBPα positively regulate each other to promote expression and maintain differentiation of 3T3-L1 cells [13]. PPARγ and C/EBPα have also been reported to induce adipogenesis upon ectopic expression in fibroblast cells [12,44,45]. In this study, ergosterol peroxide treatment (20 μM) also significantly reduced the protein expression and mRNA transcription of *PPARγ* and *C/EBPα*, which play an important role in differentiation of 3T3-L1 cells. These results suggest that ergosterol peroxide treatment decreases lipid accumulation in 3T3-L1 cells via inhibition of cell differentiation through down-regulation of *PPARγ* and *C/EBPα*.

PPARγ and C/EBPα of differentiated 3T3-L1 cells have been reported to induce the expression of *FAS*, *FAT*, *ACC*, *SCD-1*, and *SREBP-1c* genes, known as adipokines or adipogenic genes [14,15,16]. FAS catalyzes the synthesis of palmitate (C16:0, a long chain saturated fatty acid) from acetyl-CoA and malonyl-CoA, and plays an important role in regulating the chain length of released fatty acids [46,47]. FAT binds to long chain fatty acid and transports it into cells, and in vivo studies have reported that deficiency in FAT prevents obesity in patients with high-fat diets [48,49]. ACC is known as a potential target for anti-obesity drugs [50,51] and catalyzes the synthesis of malonyl-CoA, an intermediate metabolite that serves as a carbon source in the synthesis of fatty acids [52]. SCD-1 catalyzes the conversion of saturated palmitate (C16:0) and stearate (C18:0) into monosaturated palmitoleate (C16:1) and oleate (C18:1), respectively, both of which are unsaturated fatty acid constituents of triglycerides [53,54,55]. SREBP-1c is known as an important transcription factor that enhances PPAR activity by promoting the production of endogenous PPARγ ligands involved in fatty acid synthesis [17]. In this study, treatment of ergosterol peroxide (20 μM) during differentiation significantly inhibited the protein expression and mRNA transcription levels of various adipokines including FAS, FAT, ACC, and SREBP-1c. However, ergosterol peroxide did not inhibit SCD-1 protein expression and mRNA transcription. These results suggest that ergosterol peroxide does not inhibit the conversion of saturated fatty acids to unsaturated fatty acids, but inhibits triglyceride synthesis by suppressing synthesis and utilization of long chain fatty acids in 3T3-L1 cells.

During 3T3-L1 cell differentiation, proteins of the mitogen-activated protein kinase (MAPK) pathway, including extracellular signal regulatory kinase (ERK), c-jun N-terminal kinase (JNK), and p38 are activated, resulting in an increase in adipocyte number through the induction of cell proliferation [9, 10, 11]. It has also been reported that activated (phosphorylated) ERK proteins are involved in the expression of differentiation-related transcription factors, including C/EBPα, C/EBPβ, and PPARγ [11]. Inhibition of p38 has been reported to inhibit the differentiation of 3T3-L1 cells through suppression of phosphorylation-mediated activation of C/EBPβ [56,57]. Knockdown of the *JNK* gene in mouse models reared on high-fat diets increased resistance to obesity [58]. Therefore, suppression of the activation of the MAPK pathway may be helpful in preventing obesity. In this study, ergosterol peroxide was also found to inhibit the phosphorylation of ERK, JNK, and p38 in MID-treated 3T3-L1 cells. Taken together, ergosterol peroxide not only directly inhibits triglyceride synthesis in 3T3-L1 cells but also inhibits the activation of MAPK-related proteins, which play a key role in the early stage of adipocyte differentiation. These results indicate that ergosterol peroxide can be used as an effective substance for anti-obesity treatment, although further in vivo studies are needed.

## 4. Materials and Methods 

### 4.1. Purification of Ergosterol Peroxide

Crushed *G. lucidum* (4 kg) was extracted with ethanol (40 L) at room temperature for 7 days. The ethanol extracts (87 g) were evaporated, suspended in distilled water (2 L) and divided into two fractions (3 L each) with ethyl acetate as the non-aqueous phase. The ethyl acetate layer (70 g) was concentrated in vacuo, and its crude extracts were chromatographed on a silica gel (230–400 mesh, Merck Millipore, Darmstadt, Germany) by using a step gradient *n*-hexane:ethyl acetate (20:1–1:1) and chloroform:methanol (20:1–1:1) to obtain 10 fractions. The fifth and sixth fractions were combined and further separated using a silica gel (70–230-mesh, Merck Millipore, Darmstadt, Germany) column by using a step gradient chloroform:ethyl acetate (10:1–5:1) to yield four fractions. The second fraction was purified by a series of reverse phase (RP) C-18 (Merck Millipore, Darmstadt, Germany) column chromatography with acetonitrile:water (1:1–7:3) to afford ergosterol peroxide (60 mg, purity >98.0%). The structure of ergosterol peroxide was determined using spectroscopic nuclear magnetic resonance (NMR) and comparing the corresponding NMR data in literature [40].

### 4.2. 3T3-L1 Cell Cultures and Differentiation

Murine preadipocyte 3T3-L1 cells (American Type Culture Collection, Manassas, VA, USA) were cultured in DMEM (Dulbecco’s modified Eagle’s medium; Gibco BRL, Grand Island, NY, USA) supplemented with 10% NBCS (new born calf serum; Gibco BRL, Grand Island, NY, USA), 1% Pen Strep (10,000 unit/mL penicillin and 10,000 μg/L streptomycin; Gibco BRL, Grand Island, NY, USA) at 37 °C (5% CO_2_). For adipocyte differentiation, 3T3-L1 cells were incubated in DMEM containing NBCS for 48 h at 37 °C (5% CO_2_). At two days post-confluence (day 0), cells were treated with DMEM containing 10% fetal bovine serum (FBS; Gibco BRL, Grand Island, NY, USA), 0.5 mM IBMX (3-isobutyl-1-methylxanthine; Sigma-Aldrich; St. Louis, MO, USA), 1 μM dexamethasone (Sigma-Aldrich; Seoul, Korea), and 1 μg/mL insulin (Sigma-Aldrich; Seoul, Korea). After two days incubation (day 2), the medium was replaced with DMEM containing 10% FBS and 1 µg/mL insulin, and from the fourth day (day 4) the medium was replaced with DMEM containing only 10% FBS, which was changed every two days thereafter until analysis. Ergosterol peroxide (10 or 20 μM) was administered four times every two days from day 0 to day 6.

### 4.3. Cell Viability Assay

3T3-L1 cell viability was analyzed using a 3-(4, 5-dimethrylthiazol-2-yl)-2, 5-dipheyltetrazolium bromide (MTT) assay [59]. Cells were cultured in DMEM supplemented with 10% NBCS at 37 °C (5% CO_2_) for 2 days (90% confluency) and then treated with various concentrations of ergosterol peroxide (10, 20, 40, 60, 80, and 100 μM) for 48 h. After incubation, 0.5 mg/mL of MTT (Sigma-Aldrich; Seoul, Korea) solution was administered and cells were incubated for 2 h. After the reaction, the medium was removed, MTT-formazan crystals were dissolved in 1 mL of dimethyl sulfoxide (DMSO; Sigma-Aldrich; Seoul, Korea), and 200 μL were transferred to a 96-well plate. Absorbance was measured at 570 nm using a microplate reader (TECAN, Männedorf, Switzerland).

### 4.4. Oil Red O Staining Assay

After differentiation, cells were washed with phosphate-buffered saline (PBS; Gibco BRL, Grand Island, NY, USA) and fixed with 2 mL of 10% formalin (Sigma-Aldrich; Seoul, Korea) at 4 °C for 1 h. After fixation, cells were washed with PBS and stained with 2 mL of 0.35% Oil red O (Sigma-Aldrich; Seoul, Korea) at 25 °C for 1 h. After washing with distilled water, cells were examined and photographed under a light microscope, and isopropanol was added to dissolve the precipitate. The optical density at a wavelength of 540 nm was determined by a microplate reader (TECAN, Männedorf, Switzerland).

### 4.5. Western Blot Analysis

PBS-washed cell pellets were resuspended in 100 μL of radioimmunoprecipitation assay buffer (RIPA; Thermo Scientific, Seoul, Korea) containing protease inhibitor cocktail solution (GenDEPOT, Seoul, Korea) and phosphatase inhibitor cocktail solution (GenDEPOT, Seoul, Korea) and incubated at 4 °C for 30 min. The total protein concentration was determined with the Bradford assay reagent (Bio-Rad, Seoul, Korea) at a wavelength of 595 nm (TECAN, Männedorf, Switzerland). Equal amounts of protein (10 μg) were electrophoresed using sodium dodecyl sulfate–polyacrylamide gel electrophoresis (SDS-PAGE) and transferred to a polyvinylidene difluoride (PVDF) membrane (Merck Millipore, Seoul, Korea). The membrane was saturated with 5% bovine serum albumin (BSA) and incubated with 1:1000 diluted primary antibody including PPARγ, C/EBPα, FAS, SCD-1, FAT, and ACC (Cell Signaling Technology, Danvers, MA, USA) and *β*-actin (Santa Cruz Biotech, Seoul, Korea) at 4 °C for 14 h. Western blot signals were visualized using 1:5000 diluted horseradish peroxidase (HRP) conjugated secondary antibodies and developed with Amersham^TM^ ECL^TM^ prime (GE health care, UK), and then scanned using a C-Digit blot scanner (LI-COR Biosciences, Lincoln, NE, USA). Protein expression levels were quantified by Image J software (National Institutes of Health, Bethesda, MD, USA).

### 4.6. Quantitative Real-Time PCR

Total RNA was prepared from cells with TRIzol reagent (Thermo Scientific, Seoul, Korea) according to the manufacturer’s instructions. Total RNA (1 μg) and 50 μM oligo-dT primers were mixed in 17 μL of DEPC-treated water and reacted at 70 °C for 5 min. After the reaction, 2 μL of 10 mM dNTP (Takara Bio Inc., Seoul, Korea), 5 μL of 5 × RT buffer (Promega, Seoul, Korea), and 1 μL of 200 unit/μL M-MLV RTase (Promega, Seoul, Korea) were added and cDNA was synthesized by reaction at 25 °C for 5 min, at 42 °C for 60 min and at 70 °C for 15 min. Quantitative real-time PCR (qPCR) was performed using a RotorGene 6000 (Qiagen, Seoul, Korea) with reaction mixtures containing 2 × SensiFast SYBR NO-ROX kit (Bioline, Seoul, Korea), 100 ng of cDNA, and 10 pM of each primer (Table 1). After amplification, melting curve analysis was performed to verify the specificity of the reactions. The end point used in the real-time PCR quantification, Ct, was defined as the PCR threshold cycle number. The relative quantification of the target gene expression was evaluated using the ΔΔCt method [60]. The fold change of the target gene expression relative to the control (GAPDH) was calculated as the 2^−ΔΔ*C*t^ value, which was arbitrarily defined as 1.

### 4.7. Statistical Analysis 

The data are expressed as mean ± standard deviation (SD) of at least three independent experiments. Significant differences relative to controls were evaluated using a one-way analysis of variance (ANOVA), followed by Tukey’s test and Dunnet’s test using Prism (GraphPad Software Inc., La Jolla, CA, USA).

## 5. Conclusions

In this study, we evaluated the effect of ergosterol peroxide isolated from the medicinal mushroom *Ganoderma lucidum* on the differentiation and triacylglycerol synthesis of 3T3-L1 cells. Ergosterol peroxide significantly inhibited the accumulation of triglycerides in 3T3-L1 cells stimulated with MIDI. It also inhibited the mRNA expression of *SREBP-1c*, which is known to positively regulate differentiation by increasing PPARγ activity. In addition, treatment with ergosterol peroxide significantly inhibited the expression of C/EBPα and PPARγ, key factors in the differentiation of pre-adipocytes, at the protein and mRNA levels. It has also been found to inhibit the differentiation of adipocytes by inhibiting the activation of MAPK pathway-related genes, which promote the expression and activation of differentiation-related transcription factors in 3T3-L1 cells. Ergosterol peroxide treatment also inhibited the mRNA transcription of SREBP-1c, which is known to positively regulate differentiation by increasing PPARγ activity. In addition, treatment with ergosterol peroxide was shown to inhibit the expression of FAS, FAT, and ACC involved in long chain fatty acid synthesis and transport. These results suggest that ergosterol peroxide from *G*. *lucidum* may be a potential candidate for development as an anti-obesity agent since it inhibits the metabolic syndrome.

## Figures and Tables

**Table 1:** Primers used in quantitative real-time PCR.

Primer	Forward (5′–3′)	Reverse (5′–3′)
GAPDH	AAGAAGGTGGTGAAGCAGGCATC	CGAAGGTGGAAGAGTGGGAGTTG
PPARγ	TTCAGCTCTGGGATGACCTT	CGAAGTTGGTGGGCCAGAAT
C/EBPα	GTGTGCACGTCTATGCTAAACCA	GCCGTTAGTGAAGAGTCTCAGTTTG
ACC	GCGTCGGGTAGATCCAGTT	CTCAGTGGGGCTTAGCTCTG
FAS	TTGCTGGCACTACAGAATGC	AACAGCCTCAGAGCGACAAT
FAT	TAGTAGAACCGGGCCACGTA	CAGTTCCGATCACAGCCCAT
SCD1	CATCGCCTGCTCTACCCTTT	GAACTGCGCTTGGAAACCTG
SREBP-1c	ATCGCAAACAAGCTGACCTG	AGATCCAGGTTTGAGGTGGG
